# High intensity focused ultrasound (HIFU) ablation of benign thyroid nodules – a systematic review

**DOI:** 10.1186/s40349-017-0091-1

**Published:** 2017-05-17

**Authors:** Brian Hung-Hin Lang, Arnold L. H. Wu

**Affiliations:** 1 Department of Surgery, The University of Hong Kong, Hong Kong, SAR China; 2 Department of Surgery, Division of Endocrine Surgery, Queen Mary Hospital, 102 Pokfulam Road, Hong Kong, SAR China

**Keywords:** Focused ultrasound, Thyroid nodules, Thyroidectomy, High intensity focused ultrasound, Thermal ablation

## Abstract

**Background:**

With an increasing number of imaging studies being done nowadays, the number of incidentally discovered thyroid nodules is expected to rise. Although many of these nodules are small and benign in nature, some do grow and may cause pressure and/or thyrotoxic symptoms. Surgical resection has traditionally been recommended for symptomatic nodules but is associated with risk of hypothyroidism, bleeding, infection, and nerve damage. High intensity focused ultrasound (HIFU) is one of the non-surgical thermal ablation techniques that may serve as an alternative in the treatment of benign thyroid nodules. The present review is to systematically evaluate the efficacy and safety of HIFU ablation.

**Methods:**

We comprehensively searched all studies that evaluated the use of HIFU ablation as a treatment of benign thyroid nodules from Medline (PubMed) and Cochrane Library electronic databases using specific keywords. All titles identified by the search strategy were independently screened by two authors. Case reports, animal studies, editorials, expert opinions, reviews without original data and studies on pediatric population were excluded. Multiple reports of the same dataset were assessed and the most representative and updated report of a study was included.

**Results:**

Five original studies were found. All treated thyroid nodules were confirmed to be benign cytologically and either appeared solid or predominantly (>70%) solid on ultrasonography. Only one type of commercially available US-guided device with an extracorporeal probe (3 MHz) was used in all the reported treatments. No major complications including recurrent laryngeal nerve injury, skin burn or haematoma were reported in all of the studies. The overall nodule volume reduction after single session of HIFU ablation ranged between 45 and 68%, depending on nodule size and length of follow-up.

**Conclusions:**

Despite the few number of studies, our review appeared to suggest that HIFU is a safe and efficacious method of treating symptomatic benign thyroid nodules. However, larger-scale, prospective trials with longer follow-up period are indeed required to confirm this. In terms of the ablation itself, relative to other ablation techniques, there are still much room for improvements in shortening treatment duration and expanding the range of treatable nodules.

## Background

Thyroid nodules are common and can be discovered on clinical palpation in 5% of normal individuals and in 60% by high-resolution ultrasonography (US) [1, 2]. However, most of these nodules are small and often discovered incidentally while patients undergo imaging for conditions unrelated to the thyroid gland [1, 2]. Fine needle aspiration cytology (FNAC) could often confirm that the majority (>95%) of these nodules are benign [1] and given that most do not cause symptoms, no treatment would be needed except for a repeat US assessment a few years later [1–5]. Nevertheless, there is a potential for a select group of nodules to grow over time and cause local pressure symptoms like neck pain, occasional choking, dyspnea and dysphagia or hyperthyroidism. A recent multi-center, observational study has reported that up to 15% of benign nodules grow continuously in an accelerated matter while the rest remains relatively static in size [3]. From a clinical point of view, perhaps, an early identification and intervention of these growing nodules (irrespective of their symptoms) would benefit patients in the long term.

However, the choice of early intervention available has been limited. Surgical resection has traditionally been the recommended treatment for symptomatic or growing benign thyroid nodules. The revised American Thyroid Association (ATA) recommended that surgical resection (in the form of a hemithyroidectomy or a total thyroidectomy) be considered when a benign solid or predominantly solid nodule is either large-in-size (>4 cm in diameter) or is causing compressive local symptoms or clinical concern [3]. However, surgery is associated with risk of hypothyroidism, bleeding, infection, voice hoarseness from recurrent laryngeal nerve injury which may or may not be permanent [1, 6]. Moreover, surgery requires a general anesthesia and may not be feasible in some individuals because of underlying medical morbidities [1–6].

As a result, less-invasive, non-surgical techniques have to be developed [7]. This has led to the introduction of non-surgical, minimally invasive techniques like percutaneous ethanol injection (PEIT) [7, 8] and image-guided thermal ablation techniques like laser ablation therapy (LAT) and radiofrequency ablation (RFA) [9–11]. PEIT is effective in thyroid cysts and is recommended for recurrent, benign thyroid cysts [7, 8] but for solid or predominantly-solid nodules, thermal ablation techniques like LAT and RFA are generally more effective [9–11]. Studies have found these techniques could not only result in >50% nodule size reduction but also relieve pressure symptoms in many patients [9–11].

High intensity focused ultrasound (HIFU) is one of these emerging thermal ablation techniques but has been less well described in the literature. Its major advantage over other thermal techniques is that it could induce a focused thermal tissue destruction of up to 85 °C without needing needle puncture and skin penetration [12]. This technique has been successfully applied to a wide variety of benign and malignant tumors in the pancreas, prostate, bone, liver and breast [13]. However, the clinical application of HIFU for benign thyroid nodules has been somewhat limited [7].

### Pre-clinical studies on HIFU

To date, two animal studies have looked at the feasibility of using this technology to induce a well-defined lesion in a thyroid lobe, while leaving surrounding tissues un-ablated [12, 14]. The authors initially used a device that was intended for HIFU prostate treatment. In their first study, 13 thyroid lobes (mean volume, 3.4 cm3) from eight ewes were subjected in vivo to the HIFU beam. The thyroid lobes of the ewe that were excised 13 days after HIFU treatment. On microscopy, the ablated area exhibited a well-defined central necrotic area with loss of tissue architecture and colonization of the central area with polymorphonuclear leukocytes [12]. Based on these findings, the authors concluded that HIFU was an effective energy source for thyroid ablation [12, 14].

### Clinical application

The first-ever clinical study was a single center, open-label trial conducted from 2003 to 2006 by the same French group that carried out the two animal studies [15]. Together, 25 patients were treated with an US-guided HIFU device two weeks before their scheduled thyroidectomy for a benign multinodular goiter. The device used was a computer-driven system composed of an electronics cabinet, an extracorporeal probe (3 MHz frequency) mounted on a gantry and moved by stepper motors, a cooling unit, and an ultrasound-imaging scanner (7.5 MHz 128 element imaging linear array). The amount of depth-independent pulse acoustic energy received by each nodule ranged from 35 to 94 joules. Thyroid US and thyroid function were evaluated before and after treatment. Three (12.0%) patients discontinued the HIFU treatment due to intolerable pain. Among the remaining 22 patients, 16 (72.7%) had significant echogenic changes on US following the treatment. Macroscopic and histological examinations showed that all treated lesions were confined to the targeted area without affecting neighboring structures. At pathological analysis, the extent of nodule destruction ranged from 2 to 80%. In the last three patients ablated at the highest energy level, significant US changes and complete coagulative necrosis were observed in 80, 78, and 58% within the targeted area, respectively. There were no major complications of ablation ablated at the highest energy level [15]. However, despite the success of the first human study, the general adoption of this technique in routine clinical practice has been slow. To date, few clinical studies have evaluated the efficacy of HIFU treatment for benign thyroid nodules. In the United States, the use of US-guided HIFU device has been under review by the Federal and Drug Association (FDA) as a treatment option for benign thyroid nodules. Given the scarcity of evidence, the present review aimed to comprehensively evaluate the efficacy and safety of HIFU treatment of patients with benign thyroid nodule from the current literature.

## Methodology

To comprehensively review the current literature, we electronically retrieved all studies that evaluated the use of HIFU in the treatment of benign thyroid nodules from Medline (PubMed) and Cochrane Library electronic databases. The search was carried out on 27th October 2016. We used the following free text search terms in “All fields”

- #1: ‘high intensity focused ultrasound’
- #2: ‘focused ultrasound’
- #3: ‘thyroid nodule’
- #4: ‘thyroid neoplasm’
- #5: #1 OR #2 AND #3 or #4

There was no language restriction or methodological filters. The bibliographies of eligible studies were searched for other additional relevant references. All titles identified by the search strategy were independently screened by two authors (ALW, BHL). Search results were compared, and disagreements were resolved by consensus. Abstracts of potentially relevant titles were then reviewed for eligibility and full-length articles were selected for closer examination. Since there were no randomized trials, any prospective or retrospective study was included. However, case reports, animal studies, editorials, expert opinions, reviews without original data and studies on pediatric population were not included. In case of multiple reports on the same dataset, only the most representative and updated report was selected.

All data were extracted onto a standardized form. The primary data extracted from each article included number of nodules ablated by HIFU, mean size (volume) of ablated nodules, device used, amount of energy delivered per treatment, treatment time, treatment-related complications and efficacy (or % of nodule shrinkage) at any time points following treatment.

### Outcomes measured

These included treatment efficacy (i.e. extent of nodule shrinkage (%) within the first year from baseline as determined by serial US assessments), changes in symptoms and incidence of treatment-related complications.

## Results

Eleven articles were identified from our electronic search and of these, 2 were review articles [7, 16], 1 was a case report [17], 1 was an animal study [14] and 1 was an editorial [18]. Therefore, 6 articles were deemed to be original articles. Of these, 2 studies [19, 20] appeared to share similar set of patient data and so the least informative article [20] was excluded. Therefore, in the end, only 5 studies [15, 19, 21–24] were considered to contain original data. No additional study was found from our search of the bibliographies of the two review articles [7, 16]. In terms of country of origin, the first published article came from France [15]. This was followed by 3 studies from Germany [19, 21, 22], 1 study from Bulgaria [23] and 1 from Hong Kong, China [24].

### Case selection

Table 1 compares the inclusion and exclusion criteria of the 5 studies [15, 19, 21–24]. All studies included cytologically benign nodules that appeared solid or predominantly (>70%) solid on US [15, 23, 24]. Thyroid nodules were generally confirmed benign on at least one FNAC or trucut biopsy [23, 24]. Nodules that were indeterminate or suspicious of malignancy on FNAC were not treated by HIFU ablation. Limited neck extension was one of the most exclusions, because patients generally had to extend their neck during treatment [15, 23, 24]. A majority of studies only selected nodules that were causing pressure symptoms, cosmetic concern or hyperthyroidism [19, 21, 24]. Interestingly, there was no upper size limit imposed on the treated nodules. However, 2 studies did set a lower size limit at > = 10 mm [23, 24].

**Table 1:** A literature summary of HIFU studies adapting different criteria

First author (year)	Inclusion criteria	Exclusion criteria
Esnault (2011) [15]	- At least two thyroid nodules, with at least one for surgery - Nodule targeted for HIFU located at least 3 mm from the trachea, esophagus, recurrent nerve, carotid artery, skin - Selected nodule for HIFU treatment was different from the one indicated for surgery	- Suspicion of malignancy nodule, neck irradiation, previous surgery, previous radioactive iodine treatment - Any cystic components ≥ 20% or any large calcifications - Patient unable to maintain a stable position with hyperextended neck
Korkusuz (2014) [19]	At least one benign thyroid nodule with associated thyrotoxicosis, neck pain, throat hoarseness, swallowing disorders, discomfort and/or cost concern.	- Malignant nodule - Close to heat-sensitive structures like the recurrent laryngeal nerve, trachea, esophagus and carotid artery
Korkusuz (2015) [21]	- Over 18 years old - At least one benign thyroid nodule with associated issues (neck pain, hoarseness, swallowing disorders, discomfort, cosmetic concerns and/or thyrotoxicosis - Refused surgery/RIT	- Malignant nodules - Target nodules close to sensible structures such as trachea, carotid arteries - Patients who showed any contraindication to HIFU (recurrent nerve anomalies, target volumes not)
Korkusuz (2015) [22]	- Patient with symptomatic nodule - Cosmetic concerns - Refused surgery or contraindicated	- Patients with asymptomatic nodules - Nodule volume ≥ 10 mL - Histological evidence for malignancy
Kovatcheva (2015) [23]	- Over 18 years old - Presence of one or more thyroid nodules without signs of malignancy - A nodule measured on US ≥10 mm in three orthogonal dimensions - ≤30% of the targeted nodule is cystic - HIFU accessibility of the targeted nodule - Normal thyrotropin concentrations - Absence of vocal cord immobility at laryngoscopy	- Head and/or neck disease which prevents hyperextension of neck - Past medical history of thyroid cancer or other malignant tumors in the neck region - History of neck irradiation - Intra-nodular macro-calcifications which precludes treatment with HIFU - Nodules next to posterior margin of the thyroid lobe with anteroposterior diameter less than 15 mm - Pregnancy/lactation - Any contraindications related to intravenous moderate sedation
Lang (2017) [24]	- Benign cytology and low to very low suspicion sonographic pattern - Nodule believed to be causing pressure symptoms - All 3 dimensions between 10 and 40 mm - Nodule ≥ 70% solidity - Nodule within 5–30 mm from skin - Normal thyroid function and calcitonin levels	- Age ≤ 18 years old - Pregnant or lactating women - Indeterminate or malignant nodules - Intra-nodular macro-calcifications - History of head and neck irradiation - History of non-medullary thyroid carcinoma - Pre-existing vocal cord palsy

### Assessment of response

US and US Doppler were the two common treatment assessment modalities. For US Doppler, nodules were classified into type 1 (no nodular flow), type 2 (minimal flow) and 3 (significant blood flow) [19, 23]. One group also performed ^99m^Tc-pertechnetiate scintigraphy to measure the change in ^99m^TC uptake before and after HIFU treatment [19]. The authors showed that the median nodular ^99m^Tc-MIBI uptake was reduced by 35.5%, while the median uptake reduction for ^99m^Tc scintigraphy was 27% [19]. Among these 5 studies, only one study used visual analogue scale was used to monitor the change in symptoms or cosmesis after treatment [24].

### Technique and equipment

**Fig. 1:**
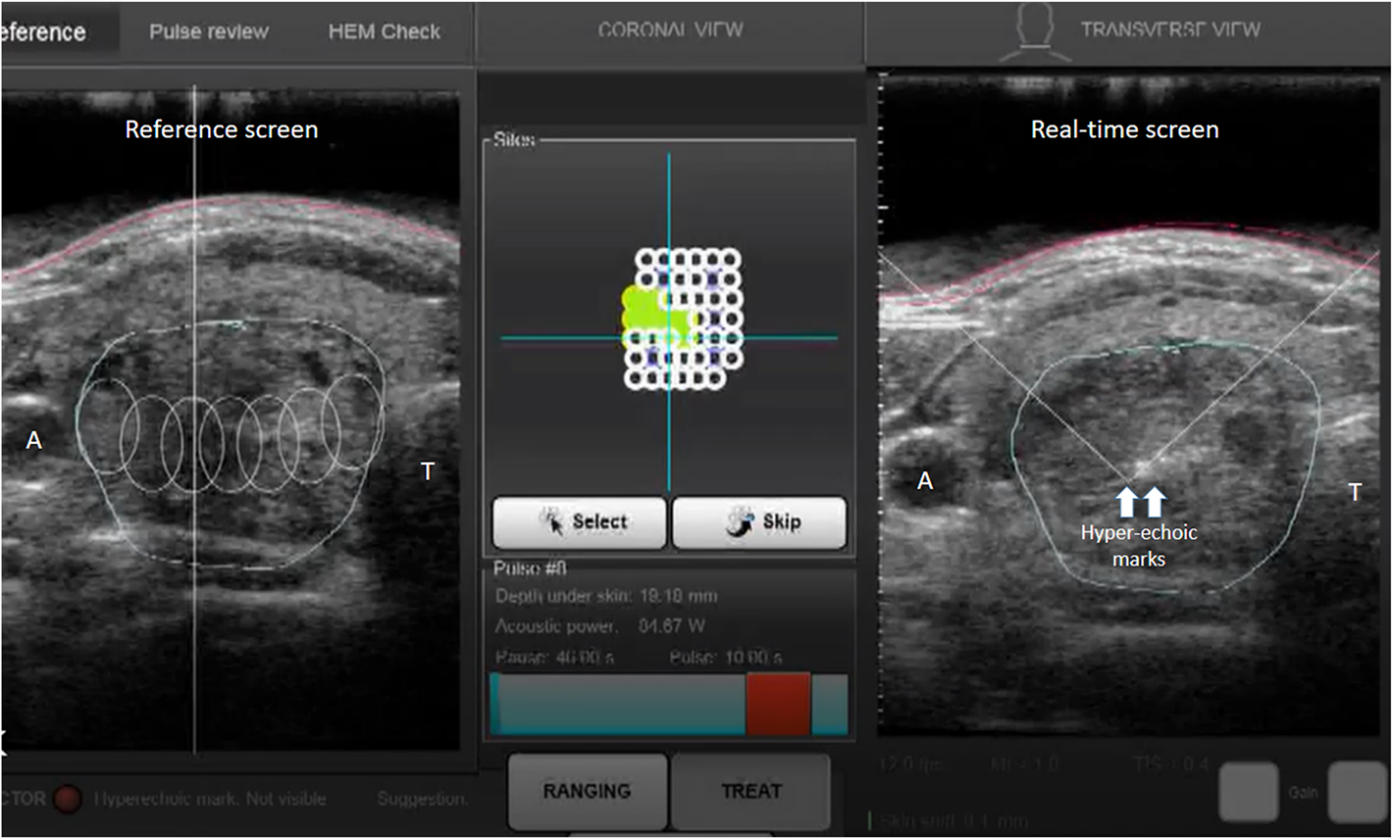
A picture of the touch-screen interface of the HIFU device. The central panel represents the birdview reconstruction of the nodule made out of multiple white cycles. The empty circles represent the unablated subunits while the filled circles represent the ablated subunits. The hyperechoic marks, on the right, are a sign of tissue necrosis from the ablationᅟ

From the literature, it seems that only one US-guided device for the treatment of thyroid nodules was available commercially [15, 19, 21–24]. This device was known as EchoPulse®(Theraclion SA, Malakoff, France). This particular device has 2 independent US systems, one for real-time imaging guidance and the other for HIFU energy delivery and treatment. Both operates via the same probe by placing it over the targeted treatment area. The US imaging system acts as a guide for the treatment system and emitts frequencies of 7.5–12 MHz (128 elements, linear array) while the treatment system has a 60 mm dimeter probe emitting 3 MHz pulses of up to 125 W of maximum acoustic power. The imaging system is placed in the midst of the probe such that the focal point of the treatment system is always displaced in the centre of the US image (Fig. 1). Hyperechoic marks signifies the presence of micro-bubbles and implies the treated area has reached a temperature above 70–80 °C (Fig. 1).

### Treatment safety and process

Given there is a number of vital heat-sensitive structures near the thyroid gland, physical limits have been set on how close the treatment beam should be during treatment. For example, the recommended safe distance for the common carotid arteries from the focus of the beam has been set at 0.2 cm, 0.3 cm for the trachea, and 0.5 cm for the recurrent laryngeal nerve. However, to date, none of these limits has been correlated with any clinical data. The Echopulse device is designed to automatically limit power on these vital areas [15, 19, 21–24]. The treatment area is planned manually into 10 to 20 sagittal and transverse section plane. Once these areas have been defined, the treatment area is further divided into voxels of 0.2 cm in diameter and 0.9 cm in length. Each voxel receives a HIFU pulse lasting over 4 to 8 s duration, raising the temperature between 60 and 80 °C, followed by a cooling period of 20 to 40 s, whilst the probe is redirected to the adjacent voxel. Cooling is provided by a cooling circulating liquid at 10 °C within a plastic balloon positioned at the forefront of the probe, preventing pre-focal tissue damage. During treatment, patients’ vital signs are monitored.

### Treatment efficacy

Table 2 shows a summary of the treatment results. Esnault et al. conducted the first clinical series of 25 patients [15]. However, since these patients underwent thyroidectomy 2 weeks after ablation, the actual extent of nodule shrinkage following HIFU could not be assessed. The authors noted that on histology, nodule destruction was observed in 22 of the 25 patients, with the remaining 3 not assessed due to insufficient number of HIFU shots received. There were no major complications except for skin blisters and pain only [15].

**Table 2:** A comparison of treatment, complications and efficacy between different HIFU studies

First author (year)	No. of nodules	Nodule volume (mL)	Type of device and probe	Total amount of DIAE to each nodule (KJ)	Treatment time (minutes)	Complications	Efficacy (% reduction from baseline)
Esnault (2011) [15]	22	0.5–2.6	US-guided HIFU/3 MHz extracorporeal probe	35–94 J/pulse^a^		Pain, skin burn, cough, blisters	Feasibility study. Ablated nodules were examined on histology after 2 weeks
Korkusuz (2014) [19]	10	Median: 3.19 (range: 0.8–7.67)	US-guided HIFU/3 MHz extracorporeal probe	Median: 8.4 (range: 5.65–12.46)	Not reported	Pain	Not reported
Korkusuz (2015) [21]	9	Median: 3.5 (range: 0.8–7.7)	US-guided HIFU/3 MHz extracorporeal probe	Median: 9.9 (range: 5.7–12.5)	Median: 62 (range: 42–96)	Pain during treatment, reddening of skin	Median: 48.8 (range: 11.4–75.0) at 3-month
Korkusuz (2015) [22]	12	Median: 3.4 (range: 0.6–5.0)	US-guided HIFU/3 MHz extracorporeal probe	-	-	None reported	Median: 55 at 3-month
Kovatcheva (2015) [23, 22]	20	Mean: 4.96 ± 2.79 (range: 1.56–9.35)	US-guided HIFU/3 MHz extracorporeal probe	Mean: 16.4 ± 7.7 (range: 5.5–31.7)	Mean: 86.8 ± 31.7 (range: 37–152)	Subcutaneous edema, skin redness	Mean: 48.7 ± 24.3 at 6-month (after single ablation)
Lang (2017) [24]	22	Mean: 6.98 ± 4.04 (range: 1.68–16.76)	US-guided HIFU/3 MHz extracorporeal probe	Mean: 15.17 ± 6.90 (range 5.88–28.35)	Mean: 75.71 ± 34.20 (range: 48.75 153.25)	Pain, skin redness, minor neck swelling	Mean: 68.87 ± 15.27 at 12-month (following single ablation)

*Abbreviations*: *US- guided HIFU* ultrasound-guided high intensity focused ultrasound, *DIAE* depth-independent acoustic energy

^a^only power was provided. Total energy was not available

Korkusuz et al. conducted an open label study on 9 patients [19]. They used a similar US-guided HIFU device with a 3 MHz probe. The mean acoustic energy used per pulse ranged from 87.6 to 192.8 joule per second. Treatment efficacy was compared with the baseline using US Doppler, ^99m^Tc scintigraphy and ^99m^Tc-MIBI scintigraphy. The nodules’ volume and blood perfusion were determined with the US Doppler. Median nodular ^99m^Tc-MIBI uptake reduced by 35.5%, while the median uptake reduction for ^99m^Tc scintigraphy was 27%. Blood perfusion was noted to decrease in 3 cases but their extent of nodule volume reduction was not documented. At 3 months, the absolute nodule volume reduction from baseline ranged from 0.4 to 4.7 ml and relative to baseline, the % of volume reduction ranged from 11.4 to 75% [11]. Although hormone level changes were not reported in the second study, Korkusuz et al. stated the in 8 of the 9 subjects, euthyrosis was achieved or preserved, with the remaining one presented with a hidden hyperthyroidism post treatment. Regarding to complications, no major complications were observed. The pain experienced during and after HIFU treatment was also recorded. On a VAS of 0 to 10, the median pain score during treatment was 5.5, whilst it was 2 at the completion of the treatment. Interestingly, instead of pain from actual treatment site, most patients reported pain spreading to surrounding areas of the throat: neck, scapula, arm, trapezius muscle [19].

Korkusuz et al*.* also looked at volume reduction, effects on thyroid function and immune response after HIFU treatment in a different study [22]. Twelve patients with an average age of 56.7 years old were treated with single-session HIFU. A volume reduction of more than 30% was recorded in 9 of the 12 patients, whilst the others did not achieve a 30% of reduction in nodule volume [22]. Triiodothyronine, thyroxine, thyrotropin and thyroglobulin were measured pre and post HIFU ablation. No significant changes were recorded at all follow-ups (24 h and 3 months) apart from thyroglobulin, which could be used to monitor damages to thyroid as well as thyroid mass [22]. Expectedly, a significant surge in thyroglobulin level was recorded 24 h after treatment but this was quickly normalized within the following 3 months.

Kovatcheva et al. measured the effects of HIFU on nodule volume at 1 week, 1 month, 3 months and 6 months on 20 patients [23]. The mean amount of depth-independent pulse acoustic energy received by each nodule was 16.4 ± 7.7 kilojoules. There was no significant difference in mean nodule volume 1 week after HIFU treatment, although it was noted that the nodule volume in 8 patients had increased by 4.7–35.1% whilst a decrease of 2.3–28.6% in 12 patients. All 20 patients recorded a decrease in mean nodular volume reduction at 1 month and 3 months, 26.3% ± 16.9 and 38.5% ± 21.6 respectively. 3 patients required a second HIFU treatment due to insufficient reduction of nodule volume (<30%) whilst another patient failed to follow-up. Volume reduction of 48.7% ± 24.3 was noted in the remaining 16 patients [23].

Recently, Lang et al*.* assessed the 6-month and 12-month efficacy of single-session of HIFU in 22 patients [24]. The mean amount of depth-independent acoustic energy to each nodule was 15.17 ± 6.90 kilojoules and the treatment time was 75.71 ± 34.20 min. Unlike previous studies, the US-guided HIFU device was updated with a new ablation software (Beamotion) which shortened ablation time while maintaining similar efficacy. Each voxel received a continuous 8-s pulse of HIFU energy followed by 40 s of cooling time before the beam moved to the adjacent voxel. This was a prospective study and had a group of patients without receiving HIFU treatment (i.e. control group). The study assessed of symptoms as a result of treatment. The authors showed that the application of HIFU ablation not only induced significant 12-month nodule shrinkage from baseline (median = 76.04%) but also improved pressure symptom score (from median = 5.0 at baseline to median = 3.0 at 6-month (*p* < 0.001) and median = 1.0; at 12-month (*p* < 0.001)) as well as improved physical composite score of Short form-12 at 12-month (median = 0.89; *p* = 0.006) [24].

**Fig. 2:**
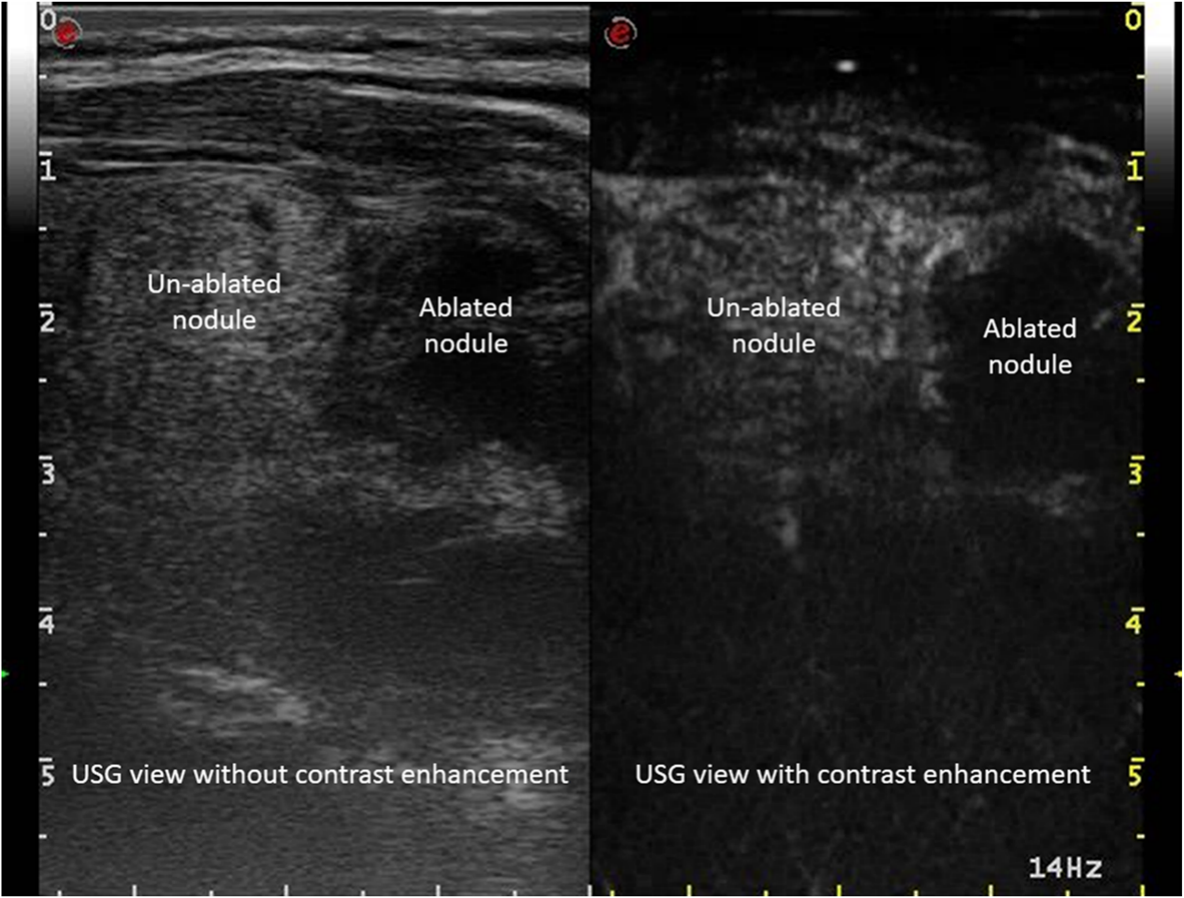
A picture showing the difference in intra-lesional contrast enhancement between an ablated and an un-ablated nodule

In addition to size shrinkage, changes in US pattern of the nodules before and after HIFU treatment has also been reported [23, 24]. For echogenicity, treated nodules tended to become more hypoechoic while for vascularization, blood flow tended to decrease over time (Fig. 2). However, these parameters are generally difficult to measure objectively.

## Discussion

HIFU ablation could be considered a truly minimally invasive procedure because it is able to induce irreversible tissue necrosis via thermal ablation beneath the skin without skin puncture or incision [7]. It has been found to be successful in the treatment of many benign and malignant tumors [13]. However, few studies have formally evaluated its use in benign thyroid nodules. In our systematic search, only 5 studies fulfilled inclusion criteria [15, 19, 21–24]. As expected, they all demonstrated the safety and feasibility of using a US-guided HIFU device to induce short-term nodule shrinkage with one study [24] even showing an improvement in local symptoms following treatment.

In terms of safety, there were no major concerns, However, it appeared pain and discomfort during treatment occurred commonly. Nevertheless, it is worth noting that the pain quickly subsided once the treatment had been completed and so, there was very little persistent pain afterwards. Skin redness and mild subcutaneous swelling were also commonly observed over the nodule area but they tended to resolve within the first 1–2 weeks. No major complications such as recurrent laryngeal nerve palsy, skin burn or haematoma were observed in any of the studies. Thyroid function seemed to be unaffected by the HIFU ablation [22].

In terms of efficacy, relative to baseline, the mean or median volume reduction ranged between 45 and 50% in the first 3 to 6 months [15, 19, 21]. However, it is worth noting that only two recent studies reported outcomes for 6 months or longer [23, 24]. The rest reported up to 3 months [15, 19, 21–24]. Consistent with the experience from other types of thermal ablation, the treated nodule tended to continually shrink over time and so, the extent of nodule shrinkage might continue for one or two years afterwards. The risk of nodule regrowth tended to be uncommon but studies with longer follow-up period are required [25]. The reported efficacies from HIFU were variable but appeared comparable to those for other thermal ablation techniques like RFA and LAT [24]. However, one should be aware that baseline nodule characteristics tended to vary between studies and so a direct comparison between techniques is impossible. Recent studies appeared to report higher rates of efficacy and this might be due to improved technique and ablation software. In terms of costs, the initial starting cost seemed to be much greater than that of other techniques, mostly due to the high start-up cost of the device (Table 3).

**Table 3:** A comparison between different thermal techniques for benign thyroid nodules

Thermal technique	Cost	Treatment duration	Main Usage
PEIT	USD 50 – USD 100	5 to 10 mins	Predominantly cystic benign thyroid nodules
RFA	Equipment: USD 25000 Consumables: USD400 per session	~15 to 30 mins	Solid functioning benign thyroid nodules
LAT	Equipment with built in laser source: ~ USD12000 Nd:YAG laser source: ~USD15,000 to USD 20,000 Consumables: ~USD400 per session	~30 mins	Cold nodules, autonomously functioning thyroid nodules and cysts
Microwave ablation	Equipment: USD 35000 Consumables: USD400 per session	~25 to 30 mins	Predominantly solid or solid benign nodules
HIFU	Equipment cost: ~USD 400,000 Annual maintenance: 10% of the base price Consumables: ~USD 350 per treatment	~60–80 min	Predominantly solid or solid benign nodules

*Abbreviations*: *PEIT* percutaneous ethanol injection therapy, *RFA* radiofrequency ablation, *LAT* laser ablation therapy, *HIFU* high intensity focused ultrasound

In terms of factors affecting treatment efficacy (as measured by extent of nodule shrinkage), although early echogenicity change, pre-ablation nodule size and vascular flow by Doppler pattern have been implicated, to date, only one study was able to find pre-ablation nodule volume as an independent significant factor. In this study, nodules ≤8.2 mL was almost two times more likely to have >50% nodule shrinkage at 12-month than nodules >8.2 mL [24].

From our systematic search, there are several shortcomings related to HIFU ablation worth highlighting. First, most of the thyroid nodules treated by HIFU had been small in size/volume when compared to studies evaluating other thermal ablation techniques [10, 11]. This is because it takes a relatively long time for a small and tight focused region to cover the entire nodule volume (Table 3). On average, using the current US-guided device, a well-selected 3 cm thyroid nodule would take approximately 45–60 min for complete ablation while for either RFA or LAT, this would be 30–40% less. Although it is possible to either increase the power or shorten the cooling period by changing the current software setting, the limiting factor with HIFU is the fact that energy still needs to propagate through the skin and deeper layers into the targeted nodule. Second, given the few number of studies, there are still many unanswered questions. For example, the efficacy of ablation remains highly unpredictable with some nodules showing a good response while others having less adequate response. Third, with the current device, treatment pulses could only apply to one single layer with a maximum treatment depth of 2.8 cm under the skin. Nodules located in deeper or more posterior part of the thyroid gland are currently not treatable with HIFU ablation.

## Conclusion

Despite the few number of studies, our review appeared to suggest that HIFU is a safe and efficacious method of treating symptomatic benign thyroid nodules. However, larger-scale, prospective trials with longer follow-up period are indeed required to further confirm this. In terms of the ablation itself, relative to other thermal ablation techniques, there are still much room for improvements in terms of treatment duration and range of treatable nodules.
